# Initial Impact of Tailored Web-Based Messages About Cigarette Smoke and Breast Cancer Risk on Boys’ and Girls’ Risk Perceptions and Information Seeking: Randomized Controlled Trial

**DOI:** 10.2196/resprot.2858

**Published:** 2013-12-10

**Authors:** Chris G Richardson, Laura L Struik, Kenneth C Johnson, Pamela A Ratner, Carolyn Gotay, Jasmina Memetovic, Chizimuzo T Okoli, Joan L Bottorff

**Affiliations:** ^1^School of Population and Public HealthUniversity of British ColumbiaVancouver, BCCanada; ^2^Institute for Healthy Living and Chronic Disease PreventionUniversity of British Columbia’s Okanagan CampusKelowna, BCCanada; ^3^Public Health Agency of CanadaOttawa, ONCanada; ^4^School of NursingUniversity of British ColumbiaVancouver, BCCanada; ^5^School of NursingUniversity of KentuckyLexington, KYUnited States

**Keywords:** breast cancer, secondhand smoke, cancer prevention, youth, gender

## Abstract

**Background:**

Recent evidence indicates a causal link between both active smoking and secondhand smoke (SHS) exposure and breast cancer (BC).

**Objective:**

The objective of the present study was to evaluate the initial reactions of girls and boys to tailored Web-based messages that describe the relationship between SHS and BC, using a parallel, single-blinded cluster randomized controlled trial.

**Methods:**

This trial was nested within a cycle of an ongoing longitudinal study of 1498 students from 74 secondary schools. Self-reported assessments were used to evaluate the impact of study messages on participants’ risk perception and interest in obtaining additional information after participants were randomized by schools to control or intervention groups. The intervention group received a tailored visual message (based on gender and Aboriginal status) about BC and tobacco smoke. The control group received a standard visual message about smoking and cancer.

**Results:**

SHS exposure was identified as a BC risk factor by 380/1488 (25.54%) participants, during the preintervention analysis. Compared to the female participants in the control group (491/839, 58.5%), girls who received the intervention (339/649, 52.2%) were 14% more likely to agree that exposure to SHS increased their BC risk (relative risk [RR] 1.14, 95% CI 1.07-1.21). Nonsmoking girls who received the intervention were 14% more likely to agree that starting smoking would increase their BC risk (RR 1.14, 95% CI 1.07-1.21). Compared to the male participants in control group (348/839, 41.5%), boys who received the intervention (310/649, 47.8%) were 10% more likely to agree that girls’ exposure to SHS increased their BC risk (RR 1.10, 95% CI 1.02-1.18). Compared to controls, girls who received the intervention were 52% more likely to request additional information about SHS and BC (RR 1.52, 95% CI 1.12-2.06).

**Conclusions:**

Brief gender-sensitive messages delivered via the Internet have the potential to increase awareness and to stimulate information seeking about the risk for BC associated with SHS.

## Introduction

### Overview

Recently published evidence indicates that there is a causal link between both active smoking and secondhand smoke (SHS) exposure and breast cancer (BC) [1]. In 2009, based on the weight of evidence from a comprehensive review of more than 100 epidemiological studies, as well as toxicology studies and an understanding of biological mechanisms, the Canadian Expert Panel on Tobacco Smoke and Breast Cancer concluded that there was a relationship consistent with causality between active smoking and BC, and between long-term regular exposure to SHS and premenopausal BC [2]. Key support for the increased premenopausal BC risk associated with SHS exposure came from a report on the health effects of environmental tobacco smoke issued by the California Environmental Protection Agency [3]. Based on a meta-analysis of 19 studies, researchers reported a relative risk (RR) of 1.25 (95% CI 1.08-1.44) for BC among all women with regular exposure to SHS [3]. This risk increased to 1.91 (95% CI 1.53-2.39) when the analysis was restricted to studies with more comprehensive SHS exposure assessment [3]. When the meta-analysis was restricted to younger, primarily premenopausal women at diagnosis, they reported RR of 1.68 (95% CI 1.31-2.15) that increased to 2.20 (95% CI 1.69-2.87) when the analysis was restricted to studies with more comprehensive SHS exposure assessment [3].

In addition to reviewing the evidence pertaining to the relationship between regular exposure to SHS and premenopausal BC, the Canadian Expert Panel on Tobacco Smoke and BC also examined findings on the relationship between active smoking and risk of BC. Key epidemiological evidence for the active smoking risk came from 8 large, high-quality cohorts studies with detailed smoking exposure measures, which indicated that early age at smoking commencement, longer duration of smoking before first birth, longer total duration, and greater number of pack-years of smoking were each associated with increased BC risk [2]. In 2011, results from the Harvard Nurses’ Health Study cohort were published based on 8772 BC cases, providing the largest and most precise analysis to date [4]. The researchers reported clear, consistent, dose-response evidence that the critical active smoking exposure period was from menarche to first full-term pregnancy, and that BC risk was limited for smoking after the first birth [4]. They also reported increasing risk-factor adjusted RRs, each statistically significant, of 11%, 19%, 21%, and 25% for 1-5, 6-10, 11-15, and ≥16 pack-years of smoking before first birth, respectively [4]. Researchers have also demonstrated that breast tissue in its growth stage, during puberty and first pregnancy, is particularly sensitive to exposure to the carcinogens found in tobacco smoke [5-7]. These findings are especially concerning given current trends in smoking initiation; the average age of smoking a whole cigarette for the first time among Canadian students in grades 6-12 is 13.4 years [8]. Moreover, given that 13% of Canadian boys between the ages of 15 and 19 years smoke, girls are at risk for SHS exposure from their male counterparts [9]. Furthermore, high rates of SHS exposure in Aboriginal communities pose particular challenges for Aboriginal girls, where Aboriginal youths’ SHS exposure is twice that of non-Aboriginal youths (27% vs 15%) [10]. To date, however, there have been few efforts to raise awareness of active smoking and SHS as risk factors for BC [11].

Research reveals that there are potential benefits in using youth-friendly approaches to deliver health information and intervention programs that are designed to change youths’ smoking behavior [12-15]. One way of addressing these preferences is by developing youth-oriented cancer control initiatives that can be delivered with interactive, socially oriented Web technologies [16-18]. Emerging evidence also indicates that tailoring tobacco control interventions toward adolescents, whereby communications are created based on adolescents’ individual characteristics, positively influences their tobacco use behavior [19]. Moreover, research has shown that developing tailored approaches for Aboriginal youth, in particular, is also beneficial [13]. For example, researchers have found that including cultural symbols (eg, feathers) in health promotion messages have been shown to signal the relevance of the health information to Aboriginal people [20].

The use of computer-based systems that facilitate the delivery of tailored interventions has been found to be an effective strategy in prompting changes in smoking behavior [21-24]. By utilizing the interactive capacity of the Internet, computer-based systems have been used by researchers to deliver tailored smoking cessation interventions according to the particular characteristics (eg, gender, cognitive variables, and intention to quit smoking) of each individual [25]. The development of tailored interventions that can be integrated into Web-based delivery systems appears to represent a potentially efficacious means of reducing adolescents’ exposure to SHS.

### Conceptual Framework

The teachable moment heuristic proposed by McBride et al [26] is conceptualized as a process of “sensemaking” of naturally occurring transitions or life events (eg, breast development in puberty) that influence people’s subjective responses to information (eg, information outlining the increased BC risk associated with SHS exposure) associated with key aspects of these transitions. These responses appear to have the potential to enhance interest in relevant information, as well as motivation to change, acquisition of skills, and self-efficacy, which in turn increase the likelihood of behavior change (eg, reductions in SHS exposure and tobacco use). Within this paradigm, perceptions of one’s personal risk play a major role in determining whether the event is significant enough to be a teachable moment that prompts the adoption of preventive health behavior [26]. Because smoking experimentation and uptake typically begin during adolescence, this early stage in boys’ and girls’ “tobacco careers” may represent a relatively malleable time to alter their tobacco use and exposure behavior. Moreover, puberty is marked with pronounced awareness of physical changes, marking girls’ transformation into womanhood [27]. These periods of heightened attentiveness to salient health transitions may enhance the cognitive availability of risk perceptions and have been identified as teachable moments for cancer prevention initiatives [26].

The delivery of messages describing the link between tobacco exposure and an increased risk of BC appears to represent an opportunity to take advantage of a naturally occurring teachable moment to promote reductions in tobacco exposure among adolescents. Within the context of cancer prevention, gender has been found to influence responses to teachable moments [28], and there is a growing body of research describing the profound influence of gender on health behavior [29]. Although gender-related factors influencing smoking initiation and patterns of exposure to tobacco have begun to be described, few attempts to develop gender-sensitive tobacco reduction interventions have been made [30].

### Primary and Secondary Hypotheses Being Tested

This study was an application of the teachable moment heuristic. The primary aim of this study was to examine youths’ responses to Web-based, gender- and Aboriginal-tailored messages regarding the link between tobacco exposure and risk of BC. We hypothesized that exposure to the tailored messages compared with a general message describing the carcinogenic aspects of tobacco smoke would result in: (1) an increased probability of indicating that tobacco exposure is associated with an increased risk of BC, and (2) an increased probability of opting to receive more information about tobacco exposure and BC. In addition to the aforementioned primary hypotheses, a secondary hypothesis that exposure to the tailored messages would be associated with more time spent viewing the messages was also tested. Each of the hypotheses was adapted to groups defined by their gender (girls and boys) and smoking status (smokers and nonsmokers).

## Methods

### Trial Design

**Figure 1:**
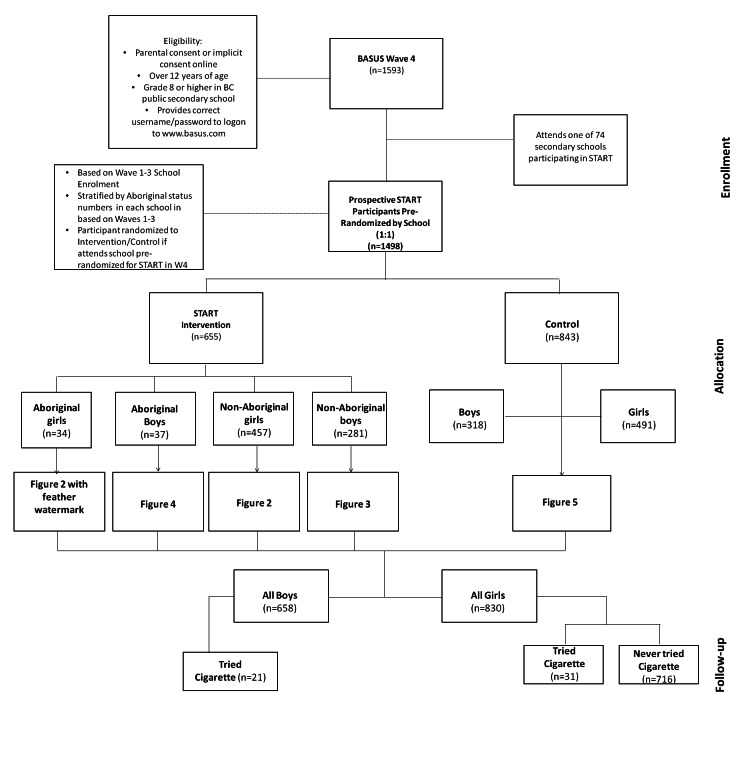
Flow diagram for START trial.

The Supporting Tailored Approaches to Reducing Tobacco (START) study was nested within the longitudinal British Columbia Adolescent Substance Use Survey (BASUS) and is a parallel, single-blinded cluster randomized controlled trial (RCT). Randomization was conducted at the school level prior to enrolment. Students were initially recruited into the BASUS study from 48 participating public secondary schools in British Columbia, Canada. All BASUS participants were 13 years of age or older, able to read and complete a Web-based survey in English, and provided informed consent, as well as written parental consent in schools requiring participants to provide parental consent. In order to prevent the enrolment of ineligible participants (eg, nonstudents), participants were recruited in person in a school environment. After viewing a brief presentation during home room class, eligible students were given an information package that contained a unique login code to set up an account on the survey website. Students completed the Web-based survey during their own time or in some cases in school computer labs during scheduled class time. Each participant received a $25 honorarium in the form of a gift card (mailed to their home address) for participating in each wave of the BASUS survey. School-specific response rates varied from 2% to 100%, with an average of 20%. For the purposes of the START study, schools (n=74) were stratified by the total number of enrolled students and number of self-identified Aboriginal students at each school (based on data from previous waves of the survey). Randomization was based on a random-number generator in MS Excel; the research manager kept the master allocation list in a password-protected computer. From April to June 2011, a subsample of 1498/1593 (94.03%) Wave 4 participants were randomized to either the intervention or the control arm, after meeting general BASUS eligibility criteria, declaring their school, and identifying their gender and Aboriginal status (Figure 1). Although researchers were not blinded to the allocation, the participants were. This study and the longitudinal BASUS study received ethics approval from the University of British Columbia Behavioral Research Ethics Board. The START study was not registered because the research team was unaware of the requirement by medical journals to register all RCTs, including those evaluating nonclinical behavioral responses to brief Web-based messages.

### Harms or Unintended Effects

There are no known harms or unintended effects to receiving either the intervention or control tailored messages.

### Data Collection

Data collection for this study occurred during Wave 4 of the BASUS survey (April to June, 2011). To reduce contamination through contact with youth in the other experimental conditions, 74 secondary schools, rather than individual participants, were randomized to receive either the control or intervention message. The participants were required to authenticate (log in) using a username and password provided by the research team. Based on their gender and ethnicity, the youth in the intervention group received a tailored message regarding BC and tobacco exposure. The control group received a standard message describing the carcinogenic aspects of tobacco smoke. Immediately after receiving the intervention or control message, participants were given follow-up questions about perceived risk and information seeking.

### Intervention

**Figure 2:**
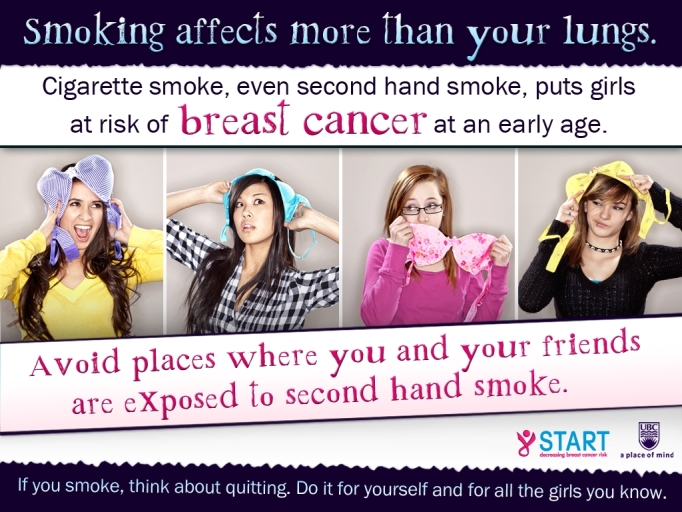
Girls' intervention message.

**Figure 3:**
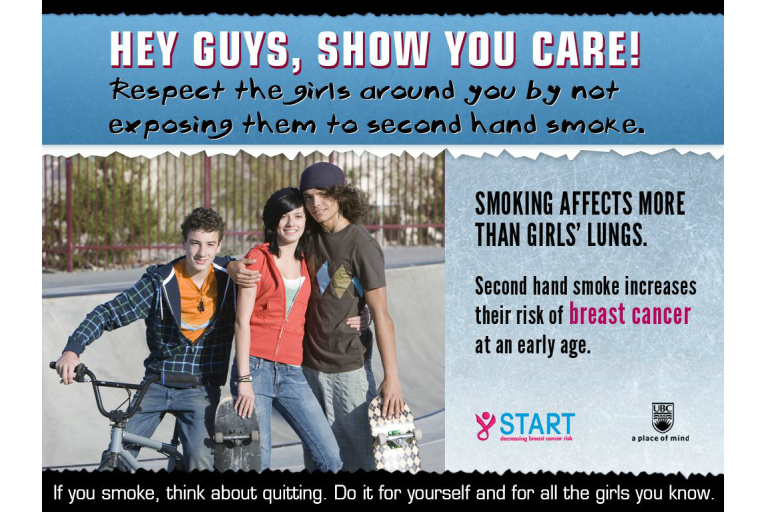
Boys' intervention message.

**Figure 4:**
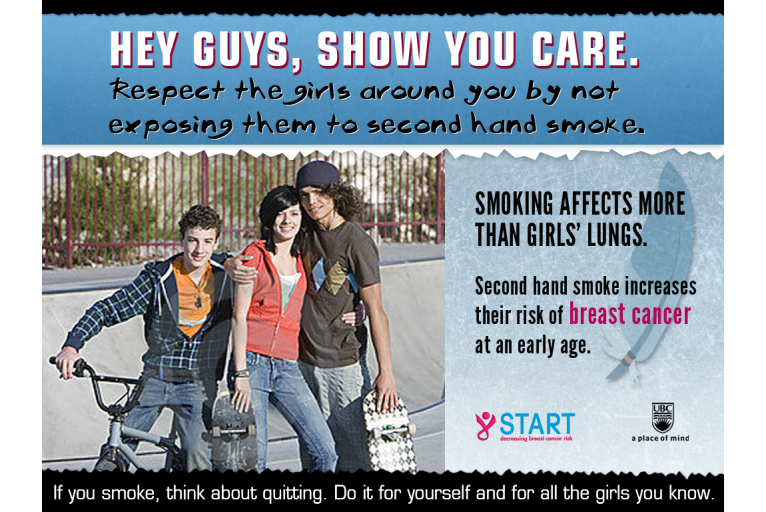
Aboriginal boys' intervention message (the difference compared to Figure 3 is the feather in the background).

The Web-based survey was programmed (ie, with the use of skip logic) so that the students in the intervention arm for each target group were presented a group-specific tailored message regarding tobacco exposure as a risk factor for BC and advice on how to minimize this risk. The development of the intervention messages was based on findings from gender- and Aboriginal-specific focus groups held with youth. We shared information about the risk of tobacco exposure and BC with the focus groups, and sought advice about the best way to communicate relevant messages to their respective target groups. Based on the focus group discussions, four messages were developed. The message for girls included images of 4 different girls playfully holding bras, with a printed message stating, “Smoking affects more than your lungs,” followed by, “Cigarette smoke, even second hand smoke, puts girls at risk for breast cancer at an early age.” The message also included a suggestion for action below the image: “Avoid places where you and your friends are exposed to second hand smoke.” The message for boys included an image of 2 boys and 1 girl standing close together in a skateboard park, with a caption stating, “Hey guys, show you care! Respect the girls around you by not exposing them to second hand smoke.” The message also included the following information: “Smoking affects more than girls’ lungs. Second hand smoke increases their risk of breast cancer at an early age.” Both the boys’ and girls’ messages included a recommendation for smokers: “If you smoke, think about quitting. Do it for yourself and for the girls you know.” Examples of the intervention messages are displayed in Figure 2 (girls’ intervention message) and Figure 3 (boys’ intervention message). The messages for the Aboriginal girls and boys were the same as the non-Aboriginal gender-sensitive messages, except for the addition of a feather motif (eg, see Figure 4). Feathers for Aboriginal people, especially eagle feathers, are ceremonial objects, used as tools for healing, and are treated with great respect [20].

### Control

**Figure 5:**
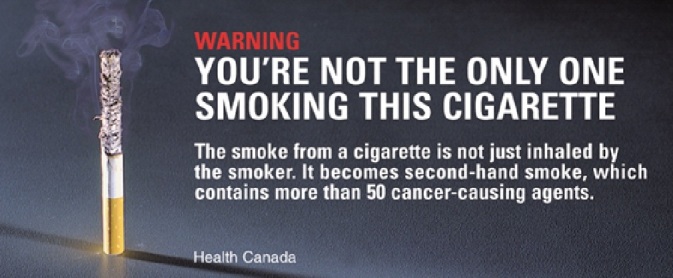
Control message.

Students in the control arm in each target group were presented with a generic gender neutral message that cigarette smoke contains carcinogenic agents. This message included an image of a burning cigarette standing alone against a black background, with the message: “Warning, you’re not the only one smoking this cigarette. The smoke from a cigarette is not just inhaled by the smoker. It becomes second hand smoke, which contains more than 50 cancer-causing agents.” This message content was sourced from Health Canada (Figure 5) [31].

### Measures

Baseline survey questions were developed to assess the participants’ sociodemographic characteristics (eg, age and ethnicity). Question topics also included smoking status, SHS exposure, and knowledge of the link between BC and tobacco. Following the presentation of the tailored intervention messages and the control messages in the survey, the youth were asked questions, tailored to their smoking status, about their perceived risk concerning tobacco exposure as a risk factor for BC. All of the girls were asked about the extent to which they agreed with the following statements: (1) “Being exposed to second hand cigarette smoke increases my risk of getting breast cancer,” and (2) “Being exposed to second hand cigarette smoke increases girls’ risk of getting breast cancer.” The girls who had already tried smoking were also asked about the extent to which they agreed with the statement: “My cigarette smoking increases my risk of getting breast cancer.” The girls who had not tried smoking were also asked about the extent to which they agreed with the statement: “If I start smoking it will increase my risk of getting breast cancer.” All of the boys were asked about the extent to which they agreed with the statement: “Being exposed to second hand cigarette smoke increases girls’ risk of breast cancer.” The boys who had tried smoking were asked about the extent to which they agreed with the statement: “Being exposed to my second hand cigarette smoke increases the breast cancer risk of the girls I spend time with.”

After the presentation of the messages and the knowledge and risk perception questions, all of the participants were asked, “Would you like to read some more information on the relationship between breast cancer and smoking?” If the participants “clicked” the answer “Yes,” they were given further information. The additional information provided to the girls included information about their risk for BC, how smoking and BC are linked, as well as strategies for reducing their risk for BC in relation to tobacco exposure. The information provided to the boys included how SHS puts girls at risk for BC, how smoking and BC are linked, as well as strategies that they could employ to protect girls from SHS exposure.

### Power Analysis

An a priori power analysis was conducted for the START study. This power analysis was based on 4 primary hypotheses for the overall START study being tested using 4 separate two-proportion z-tests to compare the knowledge of the link between cigarette smoke exposure and BC, perceptions of BC risk associated with cigarette smoke exposure, smoking behavior, and stage of change related to avoidance of SHS exposure 6 months after message delivery. Assuming a difference in proportions of 10% and a Bonferroni corrected alpha of .0125 per test (ie, alpha of .05 divided by 4), we estimated that we would need approximately 600 individuals in each group in order to have a 7 power of 0.82. It is important to note that the results presented in this paper are the initial reactions to the messages collected at baseline and not the results for the 6-month follow-up assessment for which the a priori power calculations were developed.

### Statistical Analysis

To check the potential failures in randomization, potential confounders were identified via univariate tests, and any variables found to differ significantly between the treatment and control groups were included as covariates in the subsequent multivariate models. Bivariate analyses of the categorical data were conducted using Fisher’s exact test (*P*<.05). A generalized estimating equation was used for all regression models to adjust the standard errors of the parameter estimates for the correlated responses of students within the same school [32]. Adjusted RRs were estimated using a modified Poisson regression, with robust error variance [33], originally proposed by Lee and Chia [34] for binary outcomes [35]. The robust error variance estimator was used because Poisson regression of binary outcomes tends to overestimate the standard errors [33,36]. Analyses were “intention to treat.” The statistical analysis was completed with IBM PASW Statistics 19.

## Results

### Baseline Characteristics

Of the 1593 eligible participants at baseline, 1498 (94.03%) students in 74 schools, aged 13 to 15 years (median of 14 years) participated in the current study. During the course of the study, 10 students had changed to nonstudy schools and were randomized to intervention or control groups on an individual basis. A total of 655/1498 (43.72%) students received the tailored intervention and 843/1498 (56.27%) students received the control message. Table 1 describes the participants’ baseline characteristics, patterns of tobacco exposure, and knowledge of the link between SHS and BC. The distributions of gender, age at baseline, family history of BC, intention to try smoking in the future, daily exposure to SHS in the home, as well as parents’ and peers’ smoking status were found to be significantly different between the treatment and control groups.

### Message Viewing Times in Intervention and Control Groups

The time of the initial display of the message was recorded by the survey system followed by the time of the response to the question immediately following the display of the message. The difference between these two times was treated as the message viewing time. This time includes reading and answering a single demographic question that followed the presentation of the message (ie, “How would you describe your household’s financial situation?”). Overall, the mean viewing time was 31 seconds (SD 47) for the boys and 31 seconds (SD 34) for the girls, with median viewing times of 24 seconds for the boys and 25 seconds for the girls. Both the girls and the boys in the intervention group spent significantly more time viewing the messages compared with the viewing time of the control group (girls: mean 36, SD 33 vs mean 28, SD 34, *P*<.01; boys: mean 38, SD 64 vs mean 26, SD 23, *P*<.01).

### Postintervention Perceived Risk of Tobacco Exposure

The girls that received the intervention message were 14% more likely to agree with the statement that being exposed to SHS increased their risk of BC (RR 1.14, 95% CI 1.08-1.20), and the boys were 10% more likely to agree that SHS increased the risk of BC in girls (RR 1.10, 95% CI 1.02-1.18) (see Table 2). The girls who were identified as having never tried tobacco were 14% more likely to agree with the statement that starting smoking would increase their risk of BC (RR 1.14, 95% CI 1.08-1.20). The interaction between intervention group and Aboriginal status was not significant for either boys or girls. Among the boys and girls who smoked, no significant effects were noted.

### Postintervention Information-Seeking Behavior

The girls in the intervention group were 52% more likely to seek more information, after adjusting for covariates (RR 1.52, 95% CI 1.12-2.06), compared with the control group. However, the boys in the intervention group were less likely to seek more information about the link between SHS and BC risk (RR 0.63, 95% CI 0.40-1.0); an adjusted risk ratio could not be obtained for boys likely because few had said they wanted more information (n=69).

**Table 1:** Participants’ sociodemographics and patterns of tobacco exposure.

General characteristics			Intervention (n=655)^d^ n (%)	Control (n=843)^d^ n (%)	Total (N=1498)^d^ n (%)
**Demographics**					
	**Gender** ^a^				
		Male	310 (47.80)	348 (41.48)	658 (44.22)
		Female	339 (52.23)	491 (58.52)	830 (55.78)
	**Age in years** ^c^				
		13	92 (14.18)	172 (20.50)	264 (17.74)
		14	351 (54.08)	480 (57.21)	831 (55.85)
		15	206 (31.74)	187 (22.29)	393 (26.41)
	**Ethnicity**				
		Aboriginal	71 (11.34)	96 (11.81)	167 (11.61)
		Non-Aboriginal	555 (88.66)	717 (88.19)	1272 (88.39)
	**Family income (self-reported)**				
		Below average	26 (4.24)	39 (5.01)	65 (4.67)
		Average	458 (74.71)	602 (77.38)	1060 (76.20)
		Above average	129 (21.04)	137 (17.61)	266 (19.12)
	Family history of breast cancer^a^	Yes	153 (24.60)	154 (19.59)	307 (21.80)
**Tobacco smoke exposure**					
	Has tried smoking tobacco	Yes	60 (9.20)	104 (12.40)	164 (11.00)
	**Amount smoked in lifetime (of those who tried smoking tobacco), cigarettes**				
		Had one or a few puffs	22 (38.60)	36 (35.29)	58 (36.48)
		1-5	14 (24.56)	22 (21.57)	36 (22.64)
		6-15	8 (14.04)	8 (7.84)	16 (10.06)
		16-25	1 (1.75)	8 (7.84)	9 (5.66)
		26-99	4 (7.02)	12 (11.76)	16 (10.06)
		>100	8 (14.04)	16 (15.69)	24 (15.09)
	**Age of initiation of tobacco use**				
		≤10 years old	10 (18.18)	8 (7.92)	18 (11.54)
		11 years old	5 (9.09)	10 (9.90)	15 (9.62)
		12 years old	11 (20.00)	22 (21.78)	33 (21.15)
		13 years old	13 (23.64)	40 (39.60)	53 (33.97)
		>14 years old	16 (29.09)	21 (20.79)	37 (23.72)
	**Intention to try smoking in future** ^a^ **(of those who had not tried smoking tobacco)**				
		Definitely yes	2 (0.35)	0 (0.00)	2 (0.17)
		Probably yes	12 (2.11)	30 (4.26)	42 (3.30)
		Probably not	122 (21.40)	157 (22.30)	279 (21.90)
		Definitely not	434 (76.14)	517 (73.44)	951 (74.65)
**Secondhand smoke exposure**					
	Parent(s) smoke^b^	Yes	146 (25.39)	239 (32.61)	385 (29.41)
	Friends smoke^a^	Yes	83 (17.29)	144 (23.00)	227 (20.51)
	Someone smokes in home almost every day^a^	Yes	60 (9.40)	107 (13.19)	167 (11.50)
	**Past month’s exposure to SHS**				
		Every day	20 (3.15)	35 (4.29)	55 (3.79)
		Almost every day	70 (11.04)	79 (9.68)	149 (10.28)
		At least once a week	153 (24.13)	236 (28.92)	389 (26.83)
		At least once in the past month	281 (44.32)	347 (42.50)	628 (43.31)
		Never	110 (17.35)	119 (14.58)	229 (15.79)
Tobacco knowledge: Tobacco identified as a risk factor for breast cancer		Yes	172 (26.50)	208 (24.79)	380 (25.54)

^a^ *P*<.05 (Fisher’s exact tests).

^b^ *P*<.01 (Fisher’s exact tests).

^c^ *P*<.001 (Fisher’s exact tests).

^d^Total number of responses varies slightly for each variable.

**Table 2:** Postintervention assessment of perceived risk and information seeking.

Postintervention assessments			Response^e^	Intervention, n (%)	Control, n (%)	Unadjusted RR (95% CI)	Unadjusted risk difference, %	Adjusted RR^a-d^ (95% CI)
**Increase in perceived risk of SHS**								
	My cigarette smoking increases my risk of getting BC (smoking girls) (n=32)		Agree (n=24)	8 (66.7)	16 (80.0)	0.84 (0.56-1.26)	−13.3	N/A
	If I start smoking it will increase my risk of getting BC (nonsmoking girls) (n=716)		Agree (n=659)	306 (98.4)	353 (87.2)	1.13^h^ (1.08-1.17)	11.2	1.14^h^ (1.08-1.20)
	Being exposed to secondhand cigarette smoke increases my risk of getting BC (all girls) (n=724)		Agree (n=646)	301 (95.6)	345 (84.4)	1.13^h^ (1.07-1.19)	11.2	1.14^h^ (1.07-1.21)
	Being exposed to my secondhand cigarette smoke increases the BC risk of the girls I spend time with (smoking girls) (n=31)		Agree (n=22)	9 (75.0)	13 (68.4)	1.13 (0.73-1.77)	6.6	N/A
	**Being exposed to SHS increases girls’ risk of getting BC**							
		All girls (n=720)	Agree (n=647)	303 (95.9)	344 (85.1)	1.13^h^ (1.07-1.19)	10.8	1.14^h^ (1.07-1.21)
		All boys (n=560)	Agree (n=504)	261 (93.9)	243 (87.4)	1.08^f^ (1.02-1.14)	6.5	1.10^g^ (1.02-1.18)
	Being exposed to my SHS increases the BC risk of the girls I spend time with (smoking boys) (n=21)		Agree (n=15)	7 (77.8)	8 (66.7)	1.10 (0.66-1.84)	11.1	N/A
**Interest in receiving more information**								
	All girls (n=830)		Agree (n=158)	77 (22.7)	81 (16.5)	1.37^f^ (1.04-1.82)	6.2	1.52^f^ (1.12-2.06)
	All boys (n=658)		Yes (n=69)	25 (8.1)	44 (12.6)	0.63^f^ (0.401-0.997)	−4.5	N/A

^a^Relative risk was obtained using a modified Poisson regression (with a robust covariance estimator).

^b^All models included potential confounders (age, family history of BC, intention to smoke in the future, and smoking status of parents and peers).

^c^Ethnicity (Aboriginal status) was initially included to test for an interaction with intervention group and then removed because all interactions were not significant.

^d^The model had problems with convergence due to low cell counts.

^e^ *Strongly agree* and *Agree* were collapsed as “agree” and *Strongly disagree* and *Disagree* were collapsed as “disagree,” with disagree as the referent response for the calculation of RR.

^f^ *P*<.05.

^g^ *P*<.01.

^h^ *P*<.001.

## Discussion

### Principal Findings

This is one of the first studies to evaluate the delivery of Web-based messages aimed to raise awareness about SHS exposure and BC among youth. The findings of this study indicate that the youth-informed, gender-sensitive messaging approach had positive effects on the awareness of SHS exposure as a risk factor for BC as well as on the information-seeking behavior of girls. Compared with the standard message control group, the girls who received the tailored intervention were 14% more likely to agree that being exposed to SHS increased their risk of BC. The girls who were identified as nonsmokers and received the intervention were also 14% more likely to agree that starting smoking would increase their risk of BC. Finally, compared with the girls in the control group, the girls who received the intervention were 52% more likely to request additional information about the relationship between SHS exposure and BC.

### Limitations

The findings of this study are limited in terms of their generalizability to other types of interventions, other age groups, and other ethnic groups. It is also important to note that due to sample size considerations, we elected to use a single control group that received a standard message. Larger effects would likely have been observed had we had included a third group that served as a no-information control group. Additionally, the relatively small number of Aboriginal participants and adolescents who had tried smoking at the time of the survey may have reduced the statistical power and generalizability of the results to these particular groups. The additional number of words in the tailored messages may have contributed to the finding that youth spent significantly more time viewing the tailored messages compared to the standard information control message. Despite these limitations, the results of the present study indicate that brief gender-sensitive messages delivered via the Internet have the potential to enhance awareness of the increased risk for BC associated with SHS exposure, and stimulate additional information seeking about the relationship between smoking and BC, particularly by girls. Although our messages were found to influence youths’ risk perceptions and requests for additional information, longitudinal evaluation of the intervention’s impact on health behavior (eg, reduced uptake of smoking, reduced exposure to SHS) is needed.

### Conclusions

The use of a positively framed message that promoted the benefits of being smoke free as a way to reduce the risk of BC for oneself, as well as for one’s peers, appears to be a promising approach for reaching girls. As previously suggested by Bottorff et al. [27], the juxtaposition of BC with smoking may have been particularly meaningful to girls with a growing interest in women’s health issues prompted by physical and social changes marking their transition into womanhood. This period of transition also appears to represent a teachable moment [26] that can be utilized for cancer prevention. Furthermore, the findings support the use of prevention initiatives that normalize smoke-free behavior by linking youths’ social aspirations (ie, being a good friend) with smoke-free behavior [37]. By encouraging girls to safeguard their own health, as well as the health of significant others, the tailored messages represent a promising approach to reinforcing nonsmoking girls’ smoke-free behavior.

Compared with the standard message control group, the boys who received the intervention were 10% more likely to agree that SHS exposure in girls increased their risk of BC. In addition, exposure to the tailored messages did not elicit further information seeking by the boys. The results align with literature indicating that messaging boys about a young women’s health issue is challenging because women (and girls) are traditionally expected to, and often do, look after their own health as well as the health of men rather than vice versa [38]. However, while marginal, the results indicate that a gender-sensitive approach is a promising first step toward successfully raising boys’ awareness about girls’ increased risk for BC when exposed to cigarette smoke. Awareness of the risk of SHS exposure is important for boys who smoke and may serve to motivate changes in their smoking behavior to protect girls’ health.

Adolescents frequently use the Internet to access health-related information; indeed, more than 90% of adolescents have access to the Internet at home and in school [39]. Furthermore, one-quarter of 497 adolescents recently surveyed by Ettel et al. [39] reported modifying their behavior subsequent to accessing health information on the Internet. Web-based health promotion interventions can be tailored and widely delivered to adolescents in a relatively inexpensive and effective manner. Tailoring Web-based messages according to gender, age, and ethnicity has been shown to be effective in several RCTs [40]. For example, in an RCT conducted by Mermelstein [41], adolescents who received 10 group therapy sessions with a Web-based adjunct and proactive phone calls were more likely to report smoking cessation at the 3-month follow-up compared with a control group of adolescents who received only 10 group therapy sessions. A recent meta-analysis found that compared with waitlist controls, online interventions targeting voluntary behavior change demonstrated moderate efficacy, and compared with print materials, they were equally effective but with lower costs and broader reach [42]. The findings from this study add to this body of literature in that they indicate that brief, tailored messages delivered over the Internet can be used to effectively raise awareness among youth about the risks of BC from active smoking and SHS. More generally, our application of the concept of a “teachable moment” to support the timing of this health information further supports the findings of a recent review of Web-based health promotion interventions that emphasized the importance of basing interventions on health behavior theory, including specific behavior change techniques [43].

## Multimedia Appendix 1

CONSORT-EHEALTH checklist V1.6.2 [44].

## Abbreviations

BASUS: British Columbia Adolescent Substance Use Survey
BC: breast cancer
RCT: randomized controlled trial
RR: relative risk
SHS: secondhand smoke
