# Supplementary material for: Initial Impact of Tailored Web-Based Messages About Cigarette Smoke and Breast Cancer Risk on Boys’ and Girls’ Risk Perceptions and Information Seeking: Randomized Controlled Trial
Source: JMIR Res Protoc. 2013 Dec 10;2(2):e53. doi: 10.2196/resprot.2858 (PMC3868973; doi:10.2196/resprot.2858)
Supplement: Supplementary file 1 [file resprot_v2i2e53_app1.pdf]

CONSORT-EHEALTH Checklist V1.6.2 Report

(based on CONSORT-EHEALTH V1.6), available at [http://tinyurl.com/consort-ehealth-v1-6].

2858

Date completed

10/27/2013 23:03:38

by

Chris Richardson

A randomized control trial evaluating the initial impact of receiving a tailored, web-based message on boys' and girls' perceived breast cancer risk and interest in receiving further information on the relationship between breast cancer and cigarette smoke.

TITLE

1a-i) Identify the mode of delivery in the title

Yes. The title contains the text "web-based message"

1a-ii) Non-web-based components or important co-interventions in title

Yes. There were no non-web based components to the intervention.

1a-iii) Primary condition or target group in the title

Yes. The title indicates that "boys' and girls'" are the target group.

ABSTRACT

1b-i) Key features/functionalities/components of the intervention and comparator in the METHODS section of the ABSTRACT

Yes. The abstract text indicates that:

"The intervention group received a tailored visual message (based on sex and Aboriginal status) about BC and tobacco smoke. The control group received a standard visual message about smoking and cancer."

1b-ii) Level of human involvement in the METHODS section of the ABSTRACT

Yes. The intervention consisted of viewing a single image delivered as part of a cycle of an ongoing prospective web-based survey. E.g. "The intervention group received a tailored visual message (based on sex and Aboriginal status) about BC and tobacco smoke. The control group received a standard visual message about smoking and cancer."

1b-iii) Open vs. closed, web-based (self-assessment) vs. face-to-face assessments in the METHODS section of the ABSTRACT

Yes. In the Methods section of the abstract, the recruitment is described as "This trial was nested within a cycle of an ongoing longitudinal study of 1,498 students from 74 secondary schools." Further details are provided in the body of the manuscript.

In the Objective section of the abstract, the trail is described as "a parallel, single-blinded cluster randomized control trial."

1b-iv) RESULTS section in abstract must contain use data

Yes. The results of the abstract contains the number of participants in each groups.
"Compared to controls (n=491), girls who received the intervention (n=339)" and
"Compared to controls (n=348), boys who received the intervention (n=310)"

1b-v) CONCLUSIONS/DISCUSSION in abstract for negative trials

Yes. NA - the trial results were not negative

INTRODUCTION

2a-i) Problem and the type of system/solution

Yes - see this text "The delivery of messages describing the link between tobacco exposure and an increased risk of BC appears to represent an opportunity to take advantage of a naturally occurring teachable moment to promote reductions in tobacco exposure among adolescents. Within the context of cancer prevention, gender has been found to influence responses to teachable moments [28] and there is a growing body of research describing the profound influence of gender on health behavior [29]. Although gender-related factors influencing smoking initiation and patterns of exposure to tobacco have begun to be described, few attempts to develop gender-sensitive tobacco reduction interventions have been made [30]."

and

"This study was an application of the teachable moment heuristic. The primary aim was to examine youths' responses to web-based, gender- and Aboriginal-tailored messages regarding the link between tobacco exposure and risk of BC. "

2a-ii) Scientific background, rationale: What is known about the (type of) system

Yes - see this text "The delivery of messages describing the link between tobacco exposure and an increased risk of BC appears to represent an opportunity to take advantage of a naturally occurring teachable moment to promote reductions in tobacco exposure among adolescents. Within the context of cancer prevention, gender has been found to influence responses to teachable moments [28] and there is a growing body of research describing the profound influence of gender on health behavior [29]. Although gender-related factors influencing smoking initiation and patterns of exposure to tobacco have begun to be described, few attempts to develop gender-sensitive tobacco reduction interventions have been made [30]."

METHODS

3a) CONSORT: Description of trial design (such as parallel, factorial) including allocation ratio

Yes - see this text "This study was an application of the teachable moment heuristic. The primary aim was to examine youths' responses to web-based, gender- and Aboriginal-tailored messages regarding the link between tobacco exposure and risk of BC. We hypothesized that exposure to the tailored messages compared with a general message describing the carcinogenic aspects of tobacco smoke would result in: (a) an increased probability of indicating that tobacco exposure is associated with an increased risk of BC; and (b) an increased probability of opting to receive more information about tobacco exposure and BC. In addition to the aforementioned primary hypotheses, we tested a secondary hypothesis that exposure to the tailored messages would be associated with more time spent viewing the messages. Each of the hypotheses was adapted to groups defined by their sex (girls and boys) and smoking status (smokers and non-smokers)."

3b) CONSORT: Important changes to methods after trial commencement (such as eligibility criteria), with reasons

Yes. No changes to report

3b-i) Bug fixes, Downtimes, Content Changes

Yes. No changes to report

4a) CONSORT: Eligibility criteria for participants

Yes - see this text from the Trial Design section of the Methods in the manuscript which describes eligibility criteria and recruitment procedures.

"The START study was nested within the longitudinal British Columbia Adolescent Substance Use Survey (BASUS) and is a parallel, single-blinded cluster randomized control trial. Randomization was conducted at the school level prior to enrolment. Students were initially recruited into the BASUS study from 48 participating public secondary schools in British Columbia, Canada. All BASUS participants were 13 years of age or older, able to read and complete an internet-based survey in English, and provided informed consent, as well as written parental consent in schools requiring participants to provide parental consent. In order to prevent the enrolment of ineligible participants (e.g., non-students), participants were recruited in person in a school environment. After viewing a brief presentation during home room class, eligible students were given an information package that contained a unique login code to set up an account on the survey website. Students completed the web-based survey during their own time or in some cases in school computer labs during scheduled class time. Each participant received a $25 honorarium in the form of a gift card (mailed to their home address) for participating in each wave of the BASUS survey."

4a-i) Computer / Internet literacy

Yes - see this section of text from the description of the Trial Design: "ability to read and complete the internet-based survey in English"

4a-ii) Open vs. closed, web-based vs. face-to-face assessments:

Yes - the following text describes the recruitment and assessment process:

"participants were recruited in person in a school environment. After viewing a brief presentation during home room class, eligible students were given an information package that contained a unique login code to set up an account on the survey website. Students completed the web-based survey during their own time or in some cases in school computer labs during scheduled class time. Each participant received a $25 honorarium in the form of a gift card (mailed to their home address) for participating in each wave of the BASUS survey."

4a-iii) Information giving during recruitment

Yes - see this section of the text for a description of recruitment and consent procedures:

"Students were initially recruited into the BASUS study from 48 participating public secondary schools in British Columbia, Canada. All BASUS participants were 13 years of age or older, able to read and complete an internet-based survey in English, and provided informed consent, as well as written parental consent in schools requiring participants to provide parental consent. In order to prevent the enrolment of ineligible participants (e.g., non-students), participants were recruited in person in a school environment. After viewing a brief presentation during home room class, eligible students were given an information package that contained a unique login code to set up an account on the survey website. Students completed the web-based survey during their own time or in some cases in school computer labs during scheduled class time. Each participant received a $25 honorarium in the form of a gift card (mailed to their home address) for participating in each wave of the BASUS survey. School specific response rates varied from 2% to 100%, with an average of 20%. For the purposes of the START study, schools (n=74) were stratified by total number of enrolled students and number of self-identified Aboriginal students at each school (based on data from previous waves of the survey). "

4b) CONSORT: Settings and locations where the data were collected

Yes - the abstract describes the assessments as " Self-reported assessments" in the Methods section. The Methods section of the Manuscript provides the specific wording of the self reported assessments included in the web-survey in the "Measures" section.

4b-i) Report if outcomes were (self-)assessed through online questionnaires

Yes - the abstract describes the assessments as " Self-reported assessments" in the Methods section. The Methods section of the Manuscript provides the specific wording of the self reported assessments included in the web-survey in the "Measures" section.

4b-ii) Report how institutional affiliations are displayed

Yes. We do not feel that institutional identification influenced the results of this study.

5) CONSORT: Describe the interventions for each group with sufficient details to allow replication, including how and when they were actually administered

5-i) Mention names, credential, affiliations of the developers, sponsors, and owners

Yes - The intervention consisted of single images that are provided in the Methods section. The development of the images is described in Intervention and Control sections of the Methods section of the manuscript. We do not feel that a Conflict of Interest statement is warranted given the nature of this study.

5-ii) Describe the history/development process

Yes - The intervention consisted of single images that are provided in the Methods section. The development of the images is described in Intervention and Control sections of the Methods section of the manuscript.

5-iii) Revisions and updating

Yes. There was only one final Version of the images used in this study. The development of the images is described in Intervention and Control sections of the Methods section of the manuscript.

5-iv) Quality assurance methods

Yes. The intervention consisted of single jpeg images delivered in an web-based survey. The web-survey functioning and image display properties were tested across the major internet browsers in use at the time of the web survey.

5-v) Ensure replicability by publishing the source code, and/or providing screenshots/screen-capture video, and/or providing flowcharts of the algorithms used

Yes. We have included the images (control and intervention) in the manuscript.

5-vi) Digital preservation

Yes. We have included the images (control and intervention) in the manuscript.

5-vii) Access

Yes. The images were displayed as part of an ongoing longitudinal web-based survey of secondary school students. An honorarium of $25 was provided to participants in this study.

5-viii) Mode of delivery, features/functionalities/components of the intervention and comparator, and the theoretical framework

Yes. The theoretical framework is based on the "Teachable Moment" theoretical framework. The study also integrates tailoring for gender and Aboriginal status. This is discussed in detail in the Introduction and Methods section of the manuscript.

5-ix) Describe use parameters

Yes. Does not apply to this study.

5-x) Clarify the level of human involvement

Yes. The intervention did not include any human involvement.

5-xi) Report any prompts/reminders used

Yes. This study reports the initial responses to the delivery of images displayed in a web-survey. The self-assessed responses were collected as part of the survey that contained the images so no prompts or reminders were used in this study.

5-xii) Describe any co-interventions (incl. training/support)

Yes. There were no co-interventions associated with this study.

6a) CONSORT: Completely defined pre-specified primary and secondary outcome measures, including how and when they were assessed
Yes. The following text contains our primary and secondary hypotheses.

"This study was an application of the teachable moment heuristic. The primary aim was to examine youths' responses to web-based, gender- and Aboriginal-tailored messages regarding the link between tobacco exposure and risk of BC. We hypothesized that exposure to the tailored messages compared with a general message describing the carcinogenic aspects of tobacco smoke would result in: (a) an increased probability of indicating that tobacco exposure is associated with an increased risk of BC; and (b) an increased probability of opting to receive more information about tobacco exposure and BC. In addition to the aforementioned primary hypotheses, we tested a secondary hypothesis that exposure to the tailored messages would be associated with more time spent viewing the messages."

The specific wording for the self-reported assessments are provided in the "Measures" section of the Methods of the manuscript.
6a-i) Online questionnaires: describe if they were validated for online use and apply CHERRIES items to describe how the questionnaires were designed/deployed
Yes. The assessments used in this survey were single item assessments used in the scientific literature. The specific wording for the self-reported assessments are provided in the "Measures" section of the Methods of the manuscript.
6a-ii) Describe whether and how "use" (including intensity of use/dosage) was defined/measured/monitored
Yes. We included a secondary hypothesis related to the time spent viewing the messages. See the following text from the section of the manuscript entitled Message viewing times in intervention and control groups:

"The time of the initial display of the message was recorded by the survey system followed by the time of the response to the question immediately following the display of the message. The difference between these two times was treated as the message viewing time with the recognition that it includes reading and answering a single demographic question that followed the presentation of the message (i.e., "How would you describe your household's financial situation?")."
6a-iii) Describe whether, how, and when qualitative feedback from participants was obtained
Yes. No qualitative feedback was collected for this investigation.
6b) CONSORT: Any changes to trial outcomes after the trial commenced, with reasons
Yes. There were no changes to the trial outcomes after the trial commenced.
7a) CONSORT: How sample size was determined
7a-i) Describe whether and how expected attrition was taken into account when calculating the sample size
Yes. The a priori sample size calculation for the study are reported in the manuscript under the heading Power analysis" in the Methods section of the manuscript. There was no attrition as the assessments were conducted immediately after presentation of the images during the same survey.
7b) CONSORT: When applicable, explanation of any interim analyses and stopping guidelines
Yes. This is not relevant to this study.
8a) CONSORT: Method used to generate the random allocation sequence
Yes. We describe the type of stratified randomization and process in the Trial design section of this manuscript. See the following text:

"For the purposes of the START study, schools (n=74) were stratified by total number of enrolled students and number of self-identified Aboriginal students at each school (based on data from previous waves of the survey). Randomization was based on a random-number generator in MS Excel; the research manager kept the master allocation list in a password-protected computer. Out of 1593 Wave 4 participants participating from April to June 2011, a sub-sample (n= 1,498) of participants were randomized to either the intervention or the control arm, after meeting general BASUS eligibility criteria, declaring their school, and identifying their gender and Aboriginal status (Figure 1). Although researchers were not blinded to the allocation, the participants were. "
8b) CONSORT: Type of randomisation; details of any restriction (such as blocking and block size)
Yes. Stratified randomization was used - see the following text:

"For the purposes of the START study, schools (n=74) were stratified by total number of enrolled students and number of self-identified Aboriginal students at each school (based on data from previous waves of the survey). "
9) CONSORT: Mechanism used to implement the random allocation sequence (such as sequentially numbered containers), describing any steps taken to conceal the sequence until interventions were assigned
Yes. See the following text:
"For the purposes of the START study, schools (n=74) were stratified by total number of enrolled students and number of self-identified Aboriginal students at each school (based on data from previous waves of the survey). Randomization was based on a random-number generator in MS Excel; the research manager kept the master allocation list in a password-protected computer. Out of 1593 Wave 4 participants participating from April to June 2011, a sub-sample (n= 1,498) of participants were randomized to either the intervention or the control arm, after meeting general BASUS eligibility criteria, declaring their school, and identifying their gender and Aboriginal status (Figure 1). Although researchers were not blinded to the allocation, the participants were. "
10) CONSORT: Who generated the random allocation sequence, who enrolled participants, and who assigned participants to interventions
Yes. High school students were enrolled during presentations given to them during school - usually their home room class. The presentations were given by researchers working on the study or teachers who were provided with a detailed description of the study. The random assignment was determined prior to the collection of data by a research assistant as described in the following text:

"Randomization was based on a random-number generator in MS Excel; the research manager kept the master allocation list in a password-protected computer. Out of 1593 Wave 4 participants participating from April to June 2011, a sub-sample (n= 1,498) of participants were randomized to either the intervention or the control arm, after meeting general BASUS eligibility criteria, declaring their school, and identifying their gender and Aboriginal status (Figure 1). Although researchers were not blinded to the allocation, the participants were."
11a) CONSORT: Blinding - If done, who was blinded after assignment to interventions (for example, participants, care providers, those assessing outcomes) and how
11a-i) Specify who was blinded, and who wasn't
Yes. Participants were blind to their assignment. Researchers were not blinded.
11a-ii) Discuss e.g., whether participants knew which intervention was the "intervention of interest" and which one was the "comparator"
Yes. Participants were not aware of the intervention or control status of the message they received.
11b) CONSORT: If relevant, description of the similarity of interventions
Yes. Both the control and intervention images are presented in the manuscript. Both are single images provide information on the increased risk of cancer associated with cigarette smoke exposure.
12a) CONSORT: Statistical methods used to compare groups for primary and secondary outcomes

Yes. Randomization was carried out at the school level and an adjust for this clustering was made in the analyses. See the following text:

"To check for potential failures in randomization, potential confounders were identified via univariate tests and any variables found to differ significantly between the treatment and control groups were included as covariates in the subsequent multivariate models. Bivariate analyses of the categorical data were conducted using Fisher's exact test (P value < .05). A generalized estimating equation (GEE) was used for all regression models to adjust the standard errors of the parameter estimates for the correlated responses of students within the same school [32]. Adjusted relative risks were estimated using a modified Poisson regression, with robust error variance [33], originally proposed by Lee and Chia [34] for binary outcomes [35]. The robust error variance estimator was used because Poisson regression of binary outcomes tends to overestimate the standard errors [33, 36]. Analyses were Intention to Treat. The statistical analysis was completed with IBM® PASW® Statistics 19."

12a-i) Imputation techniques to deal with attrition / missing values

Yes. There was no attrition as this study reports on data collected in the same survey that contained the intervention images.

12b) CONSORT: Methods for additional analyses, such as subgroup analyses and adjusted analyses

Yes. Analyses were adjusted for variables that were not balanced by the randomization. See the following text:

"To check for potential failures in randomization, potential confounders were identified via univariate tests and any variables found to differ significantly between the treatment and control groups were included as covariates in the subsequent multivariate models. Bivariate analyses of the categorical data were conducted using Fisher's exact test (P value < .05). A generalized estimating equation (GEE) was used for all regression models to adjust the standard errors of the parameter estimates for the correlated responses of students within the same school [32]. Adjusted relative risks were estimated using a modified Poisson regression, with robust error variance [33], originally proposed by Lee and Chia [34] for binary outcomes [35]. The robust error variance estimator was used because Poisson regression of binary outcomes tends to overestimate the standard errors [33, 36]. Analyses were Intention to Treat."

RESULTS

13a) CONSORT: For each group, the numbers of participants who were randomly assigned, received intended treatment, and were analysed for the primary outcome

Yes. These are provided in the Trial flow diagram and the results.

13b) CONSORT: For each group, losses and exclusions after randomisation, together with reasons

Yes. This is information provided in the Trial flow diagram. From the diagram it can be seen that 95 of the 1593 potential START participants logged on to the main BASUS survey but did not proceed to the START component of the survey.

13b-i) Attrition diagram

Yes.There was no attrition as this study reports on data collected in the same survey that contained the intervention images.

14a) CONSORT: Dates defining the periods of recruitment and follow-up

Yes. There is no follow-up component to this study.

14a-i) Indicate if critical "secular events" fell into the study period

Yes. This is not relevant as this study collected information on the impact of the intervention immediately after delivery of the intervention messages.

14b) CONSORT: Why the trial ended or was stopped (early)

Yes. The trial was not stopped.

15) CONSORT: A table showing baseline demographic and clinical characteristics for each group

Yes. This is provided in Table 1 of the manuscript.

15-i) Report demographics associated with digital divide issues

Yes. We report demographics associated with digital divide issues in Table 1 of the manuscript.

16a) CONSORT: For each group, number of participants (denominator) included in each analysis and whether the analysis was by original assigned groups

16-i) Report multiple "denominators" and provide definitions

Yes. The Trial flow diagram and table of results provide the necessary information.

16-ii) Primary analysis should be intent-to-treat

Yes. The analysis is based on intention-to-treat.

17a) CONSORT: For each primary and secondary outcome, results for each group, and the estimated effect size and its precision (such as 95% confidence interval)

Yes. We included 95% CI's for all effects.

17a-i) Presentation of process outcomes such as metrics of use and intensity of use

Yes. We included the testing of a secondary hypothesis that involved assessing the length of time each of the messages was viewed. This information is described in the following text taken from the manuscript:

"The time of the initial display of the message was recorded by the survey system followed by the time of the response to the question immediately following the display of the message. The difference between these two times was treated as the message viewing time with the recognition that it includes reading and answering a single demographic question that followed the presentation of the message (i.e., "How would you describe your household's financial situation?")."

17b) CONSORT: For binary outcomes, presentation of both absolute and relative effect sizes is recommended

Yes. This information is provided in Table 2 of the manuscript.

18) CONSORT: Results of any other analyses performed, including subgroup analyses and adjusted analyses, distinguishing pre-specified from exploratory

Yes. We clearly differential our primary hypotheses from the secondary hypothesis tested in the study.

18-i) Subgroup analysis of comparing only users

Yes - though this is not very relevant to this study given the nature of the intervention.

19) CONSORT: All important harms or unintended effects in each group

Yes. There were no harms or unintended effects associated with this study that we are aware of.

19-i) Include privacy breaches, technical problems

Yes. There were no privacy breaches or technical problems.

19-ii) Include qualitative feedback from participants or observations from staff/researchers

Yes. There was no qualitative feedback on the START study collected from participants.

DISCUSSION

20) CONSORT: Trial limitations, addressing sources of potential bias, imprecision, multiplicity of analyses

20-i) Typical limitations in ehealth trials

Yes. The major limitation is that we are reporting on the impact immediately following the delivery of images and the differences in risk perception may not be sustained over time. We discuss this in the recommendations for further research. We also acknowledge the low number of Aboriginal participants which limits the precision and generalizability of findings to this specific sub-population of students. This is acknowledged in the Discussion section of the manuscript.

21) CONSORT: Generalisability (external validity, applicability) of the trial findings

21-i) Generalizability to other populations

Yes. We identify a potential limitation related to the generalizability of the finding to Aboriginal youth given the relatively low number of Aboriginal participants in this study. We report a similar limitation for youth smokers. See for example the text below:

"Additionally, the relatively small number of Aboriginal participants and adolescents who had tried smoking at the time of the survey may have reduced the statistical power and generalizability of the results to these particular groups."

21-ii) Discuss if there were elements in the RCT that would be different in a routine application setting

Yes. This is not relevant to this study.

22) CONSORT: Interpretation consistent with results, balancing benefits and harms, and considering other relevant evidence

22-i) Restate study questions and summarize the answers suggested by the data, starting with primary outcomes and process outcomes (use)

Yes. For example see paragraph below from the Discussion section of the manuscript.

"The findings indicate that the youth-informed, gender-sensitive messaging approach had positive effects on the awareness of SHS exposure as a risk factor for BC as well as on the information seeking behavior of girls. Compared with the standard message control group, the girls that received the tailored intervention were 14% more likely to agree that being exposed to SHS increased their risk of BC. The girls that identified as non-smokers and received the intervention were also 14% more likely to agree that starting smoking would increase their risk of BC. Finally, compared with the girls in the control group, the girls that received the intervention were 52% more likely to request additional information about the relationship between SHS exposure and BC."

22-ii) Highlight unanswered new questions, suggest future research

Yes. For example, we explicitly identify the need for:

"longitudinal evaluation of the intervention’s impact on health behavior (e.g., reduced up-take of smoking, reduced exposure to SHS)"

Other information

23) CONSORT: Registration number and name of trial registry

No. START was not originally registered in clinicaltrials.gov due to its designation as a minimal risk study by UBC ethics, and its purpose was to examine the effect of employing a teachable moment heuristic to explore whether brief exposure to a tailored message about breast cancer and second hand smoke has immediate effects on perceived risk of SHS, knowledge about SHS and BC, and interest in receiving more information on the relationship between SHS and BC.

24) CONSORT: Where the full trial protocol can be accessed, if available

No. The trial protocol is quite simple and we feel that it is well described in the manuscript.

25) CONSORT: Sources of funding and other support (such as supply of drugs), role of funders

Yes. See text below:

"This research was supported by funding from the Canadian Breast Cancer Research Alliance and the Canadian Breast Cancer Foundation (grant # 020659), and with scholar awards from the Michael Smith Foundation for Health Research Scholar award to Dr. Oliffe, and from the Canadian Institutes of Health Research to Dr. Richardson."

X26-i) Comment on ethics committee approval

Yes. The following text is provided in the Trial Design section of the manuscript:

"This study and the longitudinal BASUS study received ethics approval from the University of British Columbia Behavioral Research Ethics Board."

x26-ii) Outline informed consent procedures

Yes. Consent from participants was obtained online by clicking continue after reading a consent screen. Consent from parents was either passive (students given information packages to take home to their parents) or active (students had to bring a signed consent form in to their school in order to be emailed a log in code for the survey. The requirement for active or passive consent varied across schools.

X26-iii) Safety and security procedures

Yes. All students were provided with a unique login code to complete the survey. All data was kept on a secure server and all personally identifiable information (e.g. name, email address or mailing address) was stored in an encrypted database that was physically separate from the survey response database.

We did not anticipate any potential harm associated with viewing the messages though information on a toll free 24 hr youth crisis line was to provided to youth as part of the information displayed on the login web page page.

X27-i) State the relation of the study team towards the system being evaluated

Yes. The manuscript explicitly identifies the researchers as creators of the intervention messages being evaluated.
